# CD160 isoforms and regulation of CD4 and CD8 T-cell responses

**DOI:** 10.1186/s12967-014-0217-y

**Published:** 2014-09-02

**Authors:** Mohamed El-Far, Charles Pellerin, Louise Pilote, Jean-Francois Fortin, Ivan A D Lessard, Yoav Peretz, Elizabeth Wardrop, Patrick Salois, Richard C Bethell, Michael G Cordingley, George Kukolj

**Affiliations:** Boehringer Ingelheim Ltd., 2100 Rue Cunard, Laval, Quebec Canada; Caprion/ImmuneCarta Services, Montreal, Québec Canada; Centre de Recherche du CHUM, Montreal, Quebec H2X 0A9 Canada; Boehringer Ingelheim (Canada) Ltd., 5180 South Service Road, Burlington, Ontario L7L 5H4 Canada

**Keywords:** CD160, PD-1, HVEM, HIV, Immunopotentiation

## Abstract

**Background:**

Coexpression of CD160 and PD-1 on HIV-specific CD8^+^ T-cells defines a highly exhausted T-cell subset. CD160 binds to Herpes Virus Entry Mediator (HVEM) and blocking this interaction with HVEM antibodies reverses T-cell exhaustion. As HVEM binds both inhibitory and activatory receptors, our aim in the current study was to assess the impact of CD160-specific antibodies on the enhancement of T-cell activation.

**Methods:**

Expression of the two CD160 isoforms; glycosylphosphatidylinositol-anchored (CD160-GPI) and the transmembrane isoforms (CD160-TM) was assessed in CD4 and CD8 primary T-cells by quantitative RT-PCR and Flow-cytometry. Binding of these isoforms to HVEM ligand and the differential capacities of CD160 and HVEM specific antibodies to inhibit this binding were further evaluated using a Time-Resolved Fluorescence assay (TRF). The impact of both CD160 and HVEM specific antibodies on enhancing T-cell functionality upon antigenic stimulation was performed in comparative *ex vivo* studies using primary cells from HIV-infected subjects stimulated with HIV antigens in the presence or absence of blocking antibodies to the key inhibitory receptor PD-1.

**Results:**

We first show that both CD160 isoforms, CD160-GPI and CD160-TM, were expressed in human primary CD4^+^ and CD8^+^ T-cells. The two isoforms were also recognized by the HVEM ligand, although this binding was less pronounced with the CD160-TM isoform. Mechanistic studies revealed that although HVEM specific antibodies blocked its binding to CD160-GPI, surprisingly, these antibodies enhanced HVEM binding to CD160-TM, suggesting that potential antibody-mediated HVEM multimerization and/or induced conformational changes may be required for optimal CD160-TM binding. Triggering of CD160-GPI over-expressed on Jurkat cells with either bead-bound HVEM-Fc or anti-CD160 monoclonal antibodies enhanced cell activation, consistent with a positive co-stimulatory role for CD160-GPI. However, CD160-TM did not respond to this stimulation, likely due to the lack of optimal HVEM binding. Finally, *ex vivo* assays using PBMCs from HIV viremic subjects showed that the use of CD160-GPI-specific antibodies combined with blockade of PD-1 synergistically enhanced the proliferation of HIV-1 specific CD8^+^ T-cells upon antigenic stimulation.

**Conclusions:**

Antibodies targeting CD160-GPI complement the blockade of PD-1 to enhance HIV-specific T-cell responses and warrant further investigation in the development of novel immunotherapeutic approaches.

**Electronic supplementary material:**

The online version of this article (doi:10.1186/s12967-014-0217-y) contains supplementary material, which is available to authorized users.

## Introduction

Negative immune regulators such as Programmed Death-1 (PD-1) and Cytotoxic T Lymphocyte Antigen 4 (CTLA-4) are part of a large network of immune checkpoints that are tightly regulated in order to limit exaggerated immune responses and prevent autoimmunity [1-4]. However, in some instances such as persistent antigenic stimulation during chronic HIV or other viral infections, these negative regulators accumulate progressively on the cell surface of total and Ag-specific T and B cells [5-9]. Expression and engagement of these negative regulators with their cognate ligands down modulate cell functions in a hierarchical manner with cell proliferation and IL-2 production being lost at earlier stages whereas IFNγ and TNFα are lost at later stages in what is referred to as immune exhaustion [10,11].

PD-1, a central negative regulatory molecule was one of the early studied mediators of immune exhaustion in chronic infectious diseases, particularly HIV-1 infection [6,7] and in animal viral chronic infectious models [12]. A large body of evidence indicates that loss of function is not simply associated with PD-1 expression alone. Other characteristics such as the level of PD-1 expression and/or its co-expression with other negative modulators may better identify functionally impaired T-cells [13,14]. Co-expression of CD160 with PD-1, 2B4 and KLRG1 on HCV-specific CD8^+^ T-cells was associated with diminished cell functions and an intermediate differentiation stage [15]. Similarly, co-expression of CD160 and PD-1 was also shown to define a subset of HIV-specific CD8^+^ T-cells with advanced dysfunction characterized by up-regulation of different inhibitory pathways and down-regulation of the NF-_Κ_B transcriptional node [14].

CD160 is a glycosylphosphatidylinositol (GPI)-anchored protein member of the Ig superfamily with a restricted expression profile that is limited to CD56^dim^ CD16^+^ NK cells, NKT-cells, γδ T-cells, cytotoxic CD8^+^ T-cells lacking the expression of CD28, a small fraction of CD4^+^ T-cells and all intraepithelial lymphocytes [16-18]. Binding of CD160 to both classical and non-classical MHC I enhances NK and CD8^+^ CTL functions [19-22]. However, engagement of CD160 by the Herpes Virus Entry Mediator (HVEM) was shown to mediate inhibition of CD4^+^ T-cell proliferation and TCR-mediated signaling [23].

HVEM protein is a bimolecular switch that binds both co-stimulatory LT-α/LIGHT and co-inhibitory receptors BTLA/CD160 (Reviewed by del Rio et al., [24]). The binding of LIGHT on T-cells to HVEM, a co-stimulatory cell surface protein expressed by immature DCs and activated T-cells, induces potent inflammatory signals and a Th1-mediated response [25]; in turn, the binding of LIGHT to HVEM on T-cells elicits activation and survival signals through the induction of NF-_Κ_B and AP1 [26,27]. In contrast, binding of HVEM to BTLA expressed by T-cells engages a potent negative signaling pathway involving both SHP-1 and SHP-2 phosphatases and effectively attenuates TCR activation [28,29]. During chronic HIV infection, *ex vivo* blockade of the HVEM network with polyclonal antibodies to HVEM enhances HIV-specific CD8^+^ T-cell functions, such as cell proliferation and cytokine production [14]. The functional effects of HVEM binding is probably influenced by several factors in addition to the interacting partner, such as cell types, strength of stimulation and expression kinetics of the receptor/ligand pairs. Consequently, the interpretation of results based exclusively on HVEM-directed blockade may benefit from additional exploration involving the interacting ligand(s).

As CD160 expression was shown to be specifically up-regulated on CD8^+^ T-cells during the chronic phase of HIV infection, we aimed in the current study to assess the targeting of CD160 receptor on HIV-specific responses. We evaluated the interaction of the two CD160 isoforms CD160-GPI and CD160-TM with HVEM ligand, as well as the impact of targeting CD160, in combination with anti-PD-1, to provide a beneficial pharmacological effect on HIV-specific CD8^+^ T-cells in response.

## Materials and methods

### Cloning of human CD160-GPI and CD160-TM isoforms

The complete CD160 cDNA sequence was synthesized *in vitro* (DNA2.0) and codon-optimized for human expression. To generate the CD160-GPI and the CD160-TM expression plasmids, the CD160 sequence was first PCR amplified using the following oligonucleotides: GATTGCAGATCTGCCACCATGCTTCTTGAACCTGGTCGCGGTTG (sense), CTGACGCTCGAGCTACAAAGCCTGCAACGCGACCAGCGAAGTTACC (antisense, CD160-GPI), CTGACGCTCGAGCTAGTGGAACTGATTCGAGGACTCTTG (antisense, CD160-TM). The PCR fragments were then digested with *Bgl*II and *Xho*I and inserted into the *Bam*HI/*Xho*I digested pcDNA3.1/neo(+) vector (Invitrogen), downstream of the CMV promoter. Note that *Bgl*II and *Bam*HI produce compatible ends.

### Production of stable cell lines

CHO-K1 (ATCC, CCL-61) stable cell lines expressing human CD160-GPI or CD160-TM were generated by lipofection of the CD160 expression vectors (pcDNA3.1) into naïve CHO-K1 cells using Lipofectamine 2000 (Invitrogen). Transfected cells were incubated at 37°C-5% CO_2_ in presence of 800 μg/ml Geneticin and, after a selection of 10–14 days, resistant T-cell colonies were isolated and transferred into 48-well tissue culture plate. Following incubation at 37°C-5% CO_2_ to allow for cell growth, cell surface expression of CD160 was evaluated with a time-resolved fluorescence assay (see below for details) using an anti-CD160 (R&D Systems, MAB6700) and an anti-mouse Eu-N1 (Perkin Elmer, AD0124). Cell clones expressing high levels of CD160-GPI or CD160-TM were expanded.

Jurkat stable cell lines expressing CD160-GPI or CD160-TM were also generated by transfecting Jurkat-NFAT-Luc cells (stably transfected with pGL4.30 NFAT-luciferase with NFAT enhancer element, Promega, and maintained with hygromycin selection) with pcDNA3.1/neo(+) vector encoding the respective CD160 isoform. The CD160-GPI form was amplified with the following PCR primers; sense: CTAGCTAGCGAGCCATGCTTCTTGAACCTGGTCGCGGTTG, anti-sense: ATAGTTTAGCGGCCGCTCACAACGCCTGCAACGCGACCAGCGAAGTTACC, and inserted into the compatible plasmid vector via the underscored *Nhe*I and *Not*I restriction sites. The CD160-TM form was PCR amplified using the CD160-GPI forward primer in combination with the following *Not*I-encoding anti-sense primer: ATAGTTTAGCGGCCGCTCACTAGTGGAACTGATTCG, and inserted into an *Nhe*I-*Not*I restricted pcDNA3.1 vector. Jurkat-CD160 positive clones were selected with Geneticin as described above.

### Time-Resolved Fluorescence (TRF) assay

A TRF assay was used to evaluate the capacity of different antibodies to inhibit the binding of recombinant human HVEM-Fc fusion protein (R&D systems, 356-HV/CF) to cells expressing either CD160-GPI or CD160-TM. In this assay, naïve CHO-K1 cells (used for background controls) or CHO-K1 cells expressing CD160 were trypsinized and diluted in F-12 media (Invitrogen) containing 10% FBS (Hyclone). Cells (40,000 per well) were then aliquoted in poly-D-lysine treated white 384-well tissue culture plates and incubated for 20 h at 37°C-5% CO_2_. After incubation, supernatant was removed and cells were washed once with 100 μl of TRF wash buffer (50 mM Tris pH 7.5, 0.05% Tween, 0.2% BSA, 150 mM NaCl). Ten μl of either CD160 or HVEM antibodies diluted in NaPO_4_ buffer (50 mM NaPO_4_ pH 6.6, 150 mM NaCl, 2% FBS) were added to each well, except for the background and the no-inhibition controls which received 10 μl of NaPO_4_ buffer, followed by the addition of 40 μl of 1.25 μg/ml HVEM-Fc, also diluted in NaPO_4_ buffer. The plate was then incubated for 1 h at RT and the wells were washed 3 times with 100 μl of TRF wash buffer. Following this wash step, 50 μl of 0.25 μg/ml anti-human Eu-N1 (Perkin Elmer, 1244–330) diluted in DELFIA assay buffer (Perkin Elmer, 1244–111) was added to each well and the plate was incubated for 1 h at RT. The wells were then washed as above (3 times 100 μl TRF wash buffer) and 50 μl of DELFIA enhancement solution (Perkin Elmer, 1244–105) were added. After an incubation of 20 min at RT, the fluorescence signal was monitored using a Wallac Victor microplate reader (excitation at 340 nm and emission at 615 nm). The antibodies tested in this assay included CD160 mAb clone CL1-R2 (MBL International), CD160 mAb clone 688327 (R&D), polyclonal anti-HVEM (R&D) and monoclonal anti-HVEM clone 94801 (R&D).

### RNA isolation from cells and quantification

The “RNeasy Kit” (Qiagen) was used to isolate RNA from cells. The total RNA concentration was determined using the “Quant-iT RiboGreen® RNA Assay Kit” from Invitrogen. The RNA concentration of the samples was determined from the standard curve generated using the ribosomal RNA standards.

### Real-time qRT-PCR assays

The “TaqMan EZ RT-PCR kit” (Applied Biosystems; ABI) was used to perform real-time (RT)-PCR reactions on a 7500 Real Time PCR System (ABI). Quantification of cellular CD160 TM RNA from primary T-cells was performed with specific primers (forward: 5’-CCCAAGCAATGAGGGTGCTATT-3’ , and reverse 5’-GGACATCCTTTCCAACCTTCTC-3’) and the 5’(FAM)-TCTGCCACCTTGGTTATTCTCCAGG-(BHQ)3’ probe (Integrated DNA Technologies; IDT). Quantification of cellular CD160-GPI RNA was performed with forward: 5’-CAACACCTTGAGTTCAGCCATA-3’; and reverse primers 5’-GACCAGCATTACCCAGACCTT-3’ and the 5’(FAM)-TGAAGGCACTCTCAGTTCAGGCTTC-(BHQ)3’ probe (IDT). The quantification of cellular CD160-GPI RNA was also performed with the “TaqMan® Gene Expression Assays” (ABI) containing gene-specific probes and primer sets. Quantification of codon-optimized CD160-GPI RNA over-expressed in Jurkat cells was performed using the following sense and anti-sense primers: 5’-GGCCATCGTGGACATTCAGT-3’; 5’-GTGCCACACCGTACAGATAAGG-3’ with a 5’(FAM)-CCGGAGGTTGCATCAACATTACAAGC-(BHQ)3’ probe. The following forward and reverse primers were used to quantify codon-optimized CD160 TM RNA: 5’-CAAGGCGGAGGAGACTGGAG-3’; 5’-GTGGAACTGATTCGAGGACTCT-3’ with the 5’(FAM)-TCACGAGGCCGGGAGAAATGTTA-(BHQ)3’ probe (IDT). The Ct values obtained for the RNA assay samples were used to interpolate an RNA copy number based on the standard curve, and the RNA copy number was normalized (by RiboGreen RNA quantification of the RNA extracted from cells and by GAPDH copy number) and expressed as quantity of copy number/μg of total RNA. The quantification of cellular GAPDH RNA transcripts was performed with the following forward and reverse primers (5’-CCTGCACCACCAACTGCTTAG-3’ , 5’-TGAGTCCTTCCACGATACCAA-3’, respectively) and the 5’(FAM)-CCCTGGCCAAGGTCATCCATG A-(BHQ)3’ probe (IDT). GAPDH RNA copy number was normalized by RiboGreen RNA quantification of the RNA extracted from cells. Serial dilutions of cellular or codon-optimized CD160-TM RNA were used to generate a standard for gene-specific expression analysis and to determine changes in transcript levels.

### Antibodies

FACS analyses used anti-CD3 (V-500), anti-CD4 (BV-605), anti-CD8 (APC-H7), anti-CD25 (A700), anti-CD134 (FITC), anti-PD-1 (eFlour 605), anti-CD45RA (ECD) anti-CCR7 (PE-Cy7), anti-CD27 (eFluor 780) and anti-CD160 clone BY55 (A647) from BD. Blocking assays used mouse monoclonal anti-CD160 clone CL1-R2 (custom purified from MBL International), mouse monoclonal anti-CD160 clone 688327, mouse monoclonal anti-HVEM clone 94801 and goat polyclonal anti-HVEM (R&D systems). PD-1 monoclonal antibody clone 5C4 (human IgG4 background) was obtained from sequence ID in patent application US20090217401; binding specificity for PD-1 and functional capacity of this antibody was characterized and confirmed (data not shown).

### Subjects

HIV-negative and HIV-1-infected subjects provided written informed consent and studies were approved by the Royal Victoria Hospital (Montreal, QC, Canada) and Boehringer-Ingelheim Institutional Review Boards. The study population of HIV subjects is shown in Table 1.

**Table 1:** **Study population and clinical characteristic of each individual HIV infected subject**

**PATIENT ID/Sex**	**Patient category**	**HLA TYPE**						**Estimated date of infection**	**VIRAL LOAD (LOG COPIES/mL PLASMA)**	**ABSOLUTE COUNT (CELLS/μL)**	
		**A**		**B**		**C**				**CD4**	**CD8**
A-KBC-1035/M	Chronic infection (No ART)*	02:02	29:02	15:03	58:02	02:10	06:02	2003	3.9	306	540
B-RPJ-1038/M	Chronic infection (No ART)	03:01	68:01	27:05	38:01	01:02	12:03	2003	2.25	289	961
C-NF-1042/M	Therapy failing	03:01	03:01	40:01	40:02	02:02	03:04	2002	4.97	393	697
D-ST-1041/M	Successfully Treated	68:01	68:01	53:01	58:02	04:01	06:02	2000	<1.7	597	1377

*ART=Anti-retroviral therapy.

### Primary cell preparation

PBMCs from subjects were obtained by leukapheresis and isolated by density gradient centrifugation (Lymphocyte Separation Medium; Wisent, St-Bruno, QC) and cryopreserved in 10% dimethyl sulfoxide (Hybri-Max DMSO; Sigma-Aldrich, St Louis, MO); 90% Heat-Inactivated Fetal Bovine Serum (HI-FBS) (PAA Laboratories, Etobicoke, ON).

### HLA typing

DNA for molecular HLA-typing was prepared from whole blood using the QIAamp DNA blood kit (Qiagen Inc., Mississauga, ON, Canada). Subjects were typed for HLA class I antigen expression (A, B, and C alleles) by sequence-based typing using kits from Atria Genetics (South San Francisco, CA). Assign software was used to interpret sequence information for allele typing (Conexio Genetics, Perth, Australia).

### Stimulation of primary CD4^+^ and Jurkat cells

Primary CD4^+^ T-cells were isolated from total PBMCs by magnetic bead separation using EasySep CD4 negative selection kit (StemCell). Purity of isolated CD4^+^ cells was consistently > 98%. Primary CD4^+^ cells were stimulated with plate-bound anti-CD3 clone UCHT1 (BD) at 1 μg/ml and anti-CD28 clone CD28.2 (BD) at 0.5 μg/ml and either human HVEM-mouse Fc fusion (R&D Systems) at a concentration of 0.2 μg/ml or its matched mouse isotype control antibody. Jurkat T-cells were activated with Dynal beads (according to the supplier’s protocol, Pan Mouse IgG, Invitrogen) coated with anti-CD3 clone UCHT1 and anti-CD28 clone CD28.2 and either anti-CD160 monoclonal antibody clone CL1-R2 (MBL International), human HVEM-mouse Fc fusion, or their matched isotype control mouse IgGs. Stimulation was performed at a ratio of 4 beads/cell.

### Tetanus toxoid stimulation assay

Total PBMCs from healthy donors were thawed in RPMI-1640 medium containing 10% heat-inactivated human serum (GemCell). Cells were washed twice with medium and suspended at a final concentration of 1.5 × 10^6^ cells/ml. Tetanus toxoid (List Biological Laboratories) was added at a concentration of 2.5 μg/ml. Blocking monoclonal antibodies against CD160, custom-purified clone CL1-R2 (MBL International) and polyclonal HVEM antibodies (R&D) or their matched isotype controls were used at 10 μg/ml. Cells were incubated for 5 to 7 days and then IFNγ was measured in the supernatant by ELISA using OptEIA Kit (BD) according to the supplier’s protocol.

### Design of peptide-pool matrices and IFNγ ELISPOT assay

The HIV peptide sets used for the CFSE and IFNγ ELISPOT assays were 15 amino acids (aa) with 11 aa overlaps. The peptides were obtained from the NIH AIDS Research and Reference Reagent Program (NARRRP, Rockville, MD). Lyophilized peptides (n = 769) spanning all HIV-1 gene products were dissolved at a concentration of 10 mg/mL in DMSO and stored at −80°C. These included 123 Gag, 249 Pol, 49 Nef, 27 Rev, 23 Tat, 46 Vif, 22 Vpr, 19 Vpu and 211 Env 15-mers corresponding to consensus clade B sequence. Pools containing 1 to 16 peptides were prepared and organized into matrices of Gag, Pol, Nef, Env and accessory (Acc) gene peptide-pools such that each peptide was present in two pools within each matrix. IFNγ secretion by HIV-specific cells was quantified using the standard ELISPOT assay. Spots were counted with the CTL ImmunoSpot 6 Analyzer (Immunospot, Cleveland, OH) and results were expressed as spot forming cells per million PBMCs (SFCs/10^6^ PBMCs) following subtraction of negative controls. The threshold for IFNγ ELISPOT positivity was set to a minimum of 50 SFC/10^6^ PBMCs following background subtraction with a minimum of 10 spots and at least two fold over background values.

### 5,6-carboxyfluorescein diacetate succinimidyl ester (CFSE) dilution assay

Thawed PBMC were resuspended in PBS 1X and labeled with 0.6 μM CFSE (Molecular Probes, Eugene, Oregon). CFSE labeled PBMCs were stimulated with 2 μg/mL of HIV consensus B peptides identified in the ELISPOT assay; Gag7876 (EKIRLRPGGKKKYKL) for subjects NF-1042 and KBC-1035, Gag937 (IYKRWIILGLNKIVR) for subject RJP-1038 and Pol5683 (TAVQMAVFIHNFKRK) for subject ST-1041, in RPMI-1640 containing 10% human AB serum (Gemini, Burlington, ON). Stimulation with media alone served as a negative control, whereas stimulation with 25 ng/ml of Staphylococcol enterotoxin B (SEB) (Sigma-Aldrich) and 2 μg/mL of CEFT (CMV, EBV, Influenza and Tetanus peptides) were used as positive control stimulations. Monoclonal antibodies directed against immune checkpoint molecules (PD-1, CD160 or HVEM) along with their corresponding isotype controls were added to the culture conditions at 5 μg/mL. All stimulatory conditions were tested in quadruplicates. Following six days of incubation at 37°C and 5% CO_2_, cells were monitored for viability with the Trypan blue exclusion test and further stained for cell surface markers using Live/Dead (Molecular Probes), αCD3, αCD8 (ebioscience), and αCD4 mAbs (BD Biosciences, Mississauga, ON). PBMCs were acquired using a BD LSRII flow cytometer and analyzed with FlowJo software version 9.4.11 (FlowJo LLC, Ashland, Oregon).

### Statistical analysis

Statistical analysis and graphical presentation was performed using GraphPad Prism 4 (GraphPad software, San Diego, CA), FlowJo 9.1 (Treestar) and FACSDiva V6 (BD Biosciences). Two-tailed paired *t* test was used to assess differences in the relative frequency of CD4^+^CD160^+^ T-cells before and after TCR stimulation from the same donors and in the IL-2 production following triggering with HVEM-Fc. The non-parametric Kruskal-Wallis and Dunn’s tests were used to analyze data on the enhancement of T cell activation as shown in Figure legends.

## Results

### Expression of CD160 isoforms on primary T-cells and binding to HVEM

**Figure 1:**
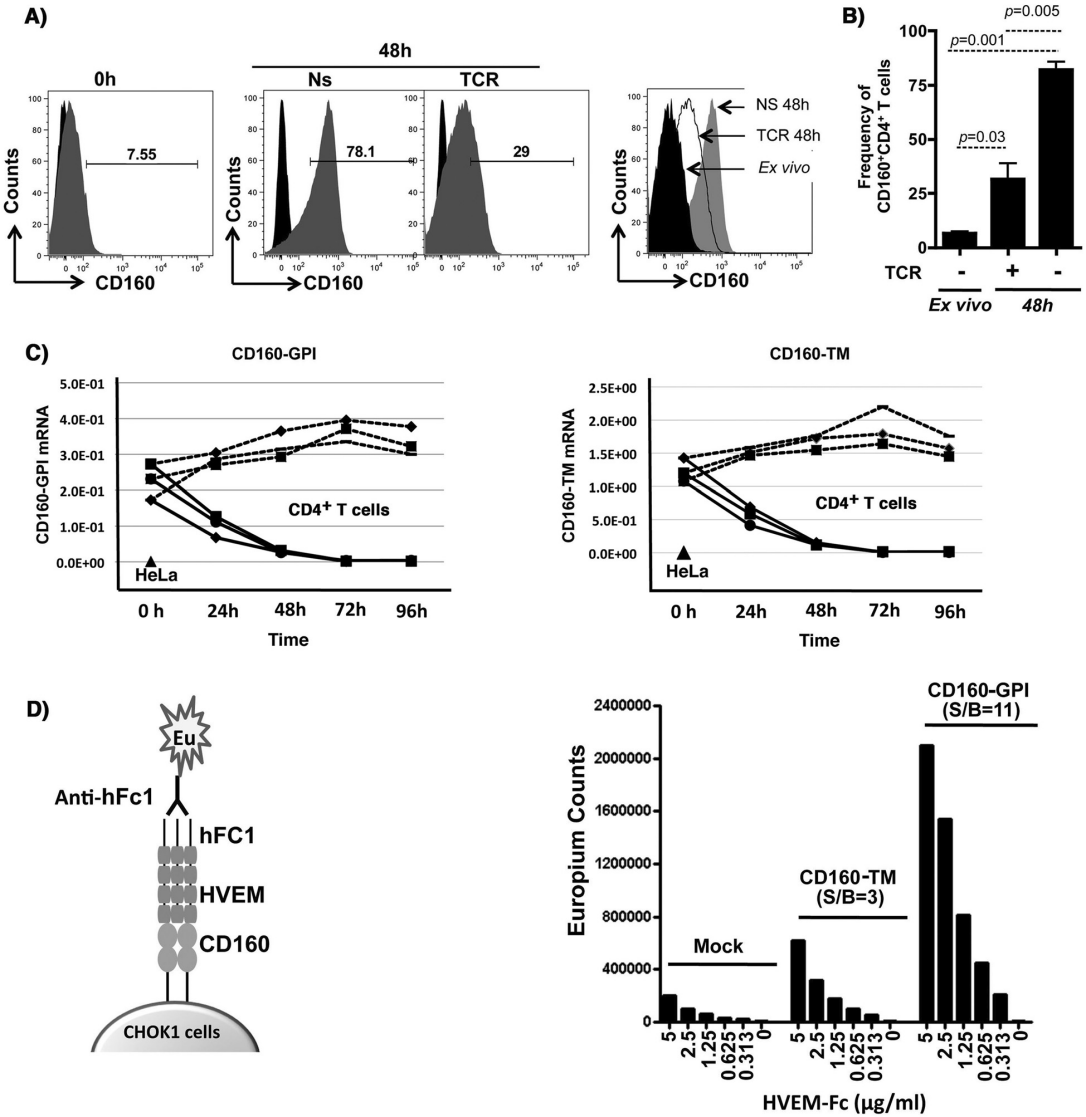
**Expression of CD160 isoforms in primary CD4** ^**+**^ **T-cells and binding to HVEM. A) Left panel:** Representative FACS analysis of CD160 on primary CD4^+^ T-cells isolated from total PBMCs of a healthy donor (*ex vivo* at baseline), gated on CD3^+^CD4^+^CD8^−^ cells. **Middle panels**: CD160 surface expression following 48 h of resting (non-stimulated, NS) or TCR activation (plate-bound anti-CD3 and soluble anti-CD28). **Right panel**: overlapping histograms showing CD160 surface expression from TCR-stimulated CD4^+^ T-cells (dotted empty histogram) in comparison to 48 h rested CD4 (filled grey histogram) and freshly isolated CD4 cells (filled black histograms) all from the same individual donor. **B)** Frequency of CD160^+^CD4^+^ double positive population following 48 h of resting or TCR stimulation compared to freshly isolated (*ex vivo*) cells (n = 3). **C)** Kinetics of CD160-GPI and CD160-TM isoform expression at the mRNA level by quantitative RT-PCR in primary CD4^+^ T-cells (cells from n = 3 independent healthy donors) stimulated through TCR for 4 days. Values are relative to the house-keeping GAPDH gene transcripts (n = 3). HeLa cells were used as a negative control for CD160 TM transcription. **D)** Binding of HVEM to the two isoforms CD160. **Left panel:** Schematic representation for the TRF binding assay between CD160 (over-expressed by CHO-K1 cells) and the soluble ligand HVEM containing the human Fc1 (detection with anti-human Fc1). **Right panel:** Measuring the signal/background (S/B) for HVEM binding to both CD160-GPI and CD160-TM cells by the TRF assay under decreasing concentrations of HVEM-Fc.

One aim of this study was to develop screening assays to evaluate the impact of CD160 antibodies on the enhancement of HIV-specific CD8 T-cell responses. CD160 was previously reported to mediate a co-stimulatory role on CD8^+^ T-cell activation upon binding to MHC-I, or a co-inhibitory role on CD4^+^ T-cell activation upon binding to HVEM. Our first aim was to establish an inhibitory assay to test anti-CD160 antibody candidates with potential blocking capacity on T-cell activation, herein CD4^+^ T-cells. To this end, we assessed the expression of CD160 on CD4^+^ T-cells before and after TCR activation to select the optimal time point for CD160 triggering. Levels of CD160 surface expression were determined using the BY55 clone of anti-CD160 that preferentially recognizes the GPI isoform [18]. Consistent with earlier reports [23], we observed that CD160 was expressed on a small fraction (2-8%) of *ex vivo* CD4^+^ T-cells at baseline (Figure 1A & B). CD160 expression on cells stimulated with anti-CD3 and anti-CD28 monoclonal antibodies was higher at 48 h post-stimulation (*p* = 0.03) compared to the *ex vivo* baseline levels. Notably, T-cells which remained un-stimulated for 48 hr showed the highest levels of CD160 compared to TCR-stimulated and *ex vivo* stained cells from matching individual donors (n = 3, *p* = 0.005 and *p* = 0.001, respectively) (Figure 1A, middle and right panels). Similar results were also obtained with CD8^+^ T-cells (data not shown). The up-regulation of CD160 on resting cells *ex vivo* and its down-regulation following TCR stimulation thus contrasted observations by Cai et al. [23] who showed that CD160 is upregulated on CD4 T-cells following TCR stimulation. Therefore, we assessed whether this discrepancy was attributable to the expression of the newly identified isoform of CD160, the full-length trans-membrane isoforme (CD160-TM). The CD160-TM isoform is induced on NK cells upon stimulation with a panel of cytokines including IL-2, IL-12, IL-15 and IL-18 [18]. Our data in Figure 1C showed that the CD160-TM isoform was indeed clearly detectable at the transcriptional level in CD4^+^ T-cells as measured by quantitative RT-PCR. However, following TCR stimulation, both CD160-TM and CD160-GPI transcripts decreased gradually with time and became undetectable by 72–96 h post-TCR stimulation. Of note, we could not confirm the specific expression of CD160-TM at the protein level due to the lack of specific antibodies capable of distinguishing between the two isoforms (note that CD160-GPI antibodies poorly recognize the CD160-TM isoform [18]).

HVEM expressed on the surface of antigen presenting cells was previously shown to bind CD160-GPI on CD4^+^ T-cells to elicit a potent inhibitory signal [23]. In order to study the details of HVEM binding to CD160 and examine its binding to the newly identified isoform of CD160-TM, we established CHO-K1 cell lines that over-expressed either CD160-GPI or CD160-TM to assess the binding of soluble HVEM-Fc. The assay was based on the highly sensitive dissociation-enhanced lanthanide fluorescent immunoassay” (DELFIA; Perkin Elmer) with time-resolved fluorescence (Figure 1D, left panel). HVEM-Fc specifically but differentially bound to the CD160-GPI and CD160-TM isoforms with a signal to background ratio (S/B) of 11 and 3, respectively (Figure 1D, right panel). Together, CD160-TM, similar to CD160-GPI, was expressed by T-cells and recognized by HVEM, albeit with a lower level of binding compared to CD160-GPI. However, this lower level of binding could be due in part to a lower surface expression of CD160-TM.

### Antibody-mediated specific blockade of CD160/HVEM binding

**Figure 2:**
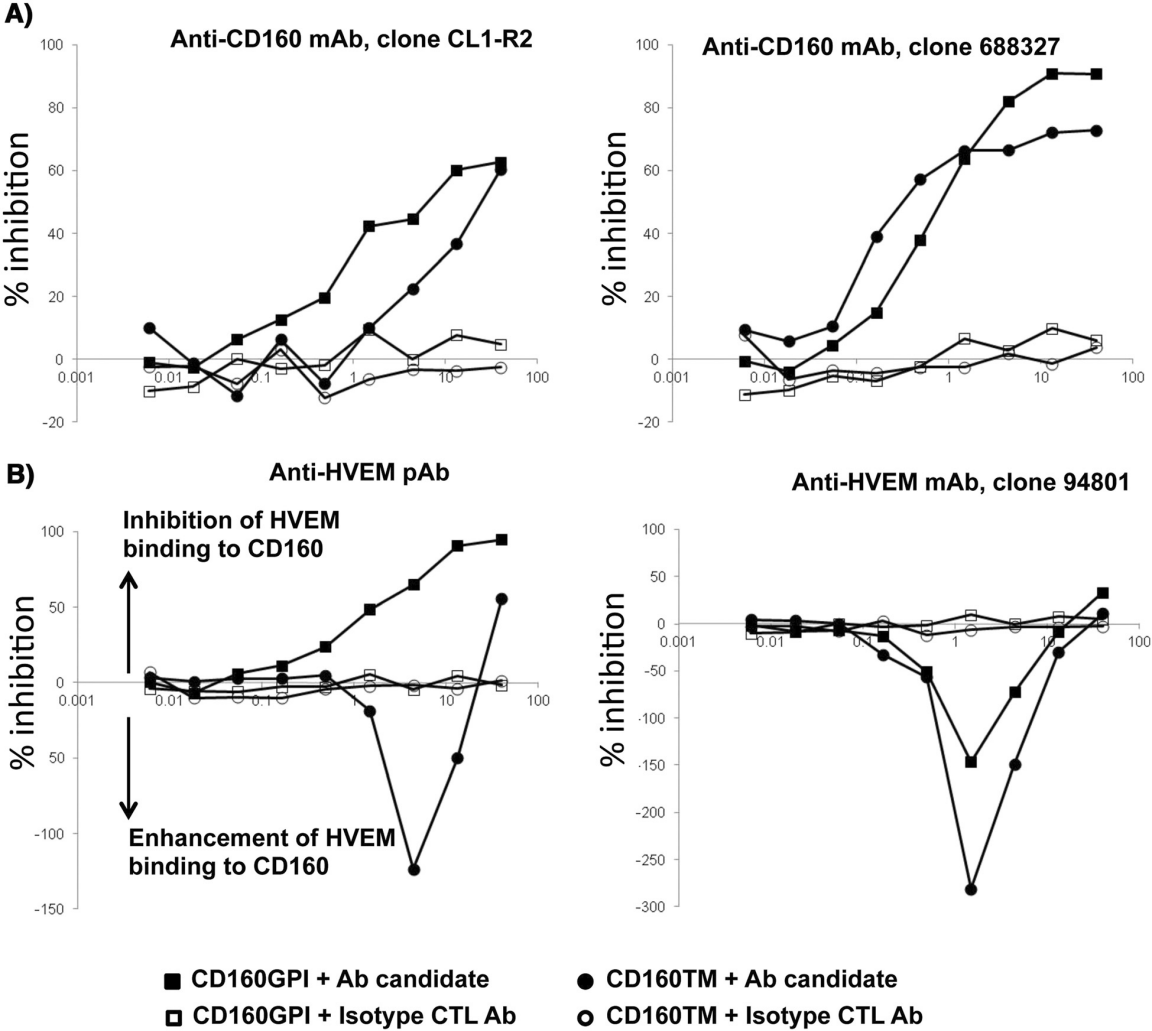
**Differential inhibition of HVEM/CD160 binding with benchmark tool antibodies.** TRF assay measuring the potency of different antibodies to inhibit the binding of recombinant human HVEM-Fc chimera to CD160^+^ CHO-K1 cells. **A)** CD160 monoclonal antibodies inhibit binding of HVEM-Fc to both CD160-GPI and CD160-TM isoforms. **B)** Polyclonal HVEM (left panel) and monoclonal HVEM (right panel) antibodies both enhance binding of HVEM-Fc to CD160-TM isoform. The polyclonal anti-HVEM inhibits HVEM-Fc binding to CD160-GPI (left panel). Antibody concentrations are plotted on the X axis whereas, the calculated percentage of inhibition of binding is plotted on the Y axis. Matched isotype control antibody for each individual antibody candidate was also used in the assay (empty circles and squares). CTL = control, mAb = monoclonal antibody, pAb = polyclonal antibody.

We next screened benchmark antibodies directed against CD160 and HVEM to evaluate their potential capacity to block CD160/HVEM interaction and to select candidates for functional rescue of antigen-specific T-cells. Previous studies have shown that binding of HVEM to CD160 can be inhibited by the CD160 monoclonal antibody (mAb) CL1-R2 [30], an antibody with antiangiogenic activity [31]. We used the TRF CD160/HVEM binding assay to confirm these observations and to further evaluate other CD160 and HVEM antibodies (some of which were previously shown to enhance HIV-specific responses, [14]). The TRF assay consisted of a fixed concentration of soluble HVEM-Fc (1 μg/ml) and serial dilutions of either CD160 mAbs (clones CL1-R2 and clone 688327) or HVEM polyclonal and monoclonal (clone 94801) Abs. CD160 Abs readily inhibited the binding of HVEM to either CD160-GPI or CD160-TM isoforms (Figure 2A) in the TRF assay. In contrast, the polyclonal HVEM antibody, which inhibited the binding of HVEM to CD160-GPI, enhanced HVEM binding to CD160-TM. Furthermore, the monoclonal HVEM Ab (clone 94801) enhanced the binding of HVEM to both CD160 isoforms (Figure 2B). Together, these results showed that CD160 or HVEM antibodies had differential capacities to inhibit (or augment) the interaction between HVEM and specific CD160 isoforms.

### Triggering of CD160-GPI is consistent with positive regulation of CD4^+^ T-cells

**Figure 3:**
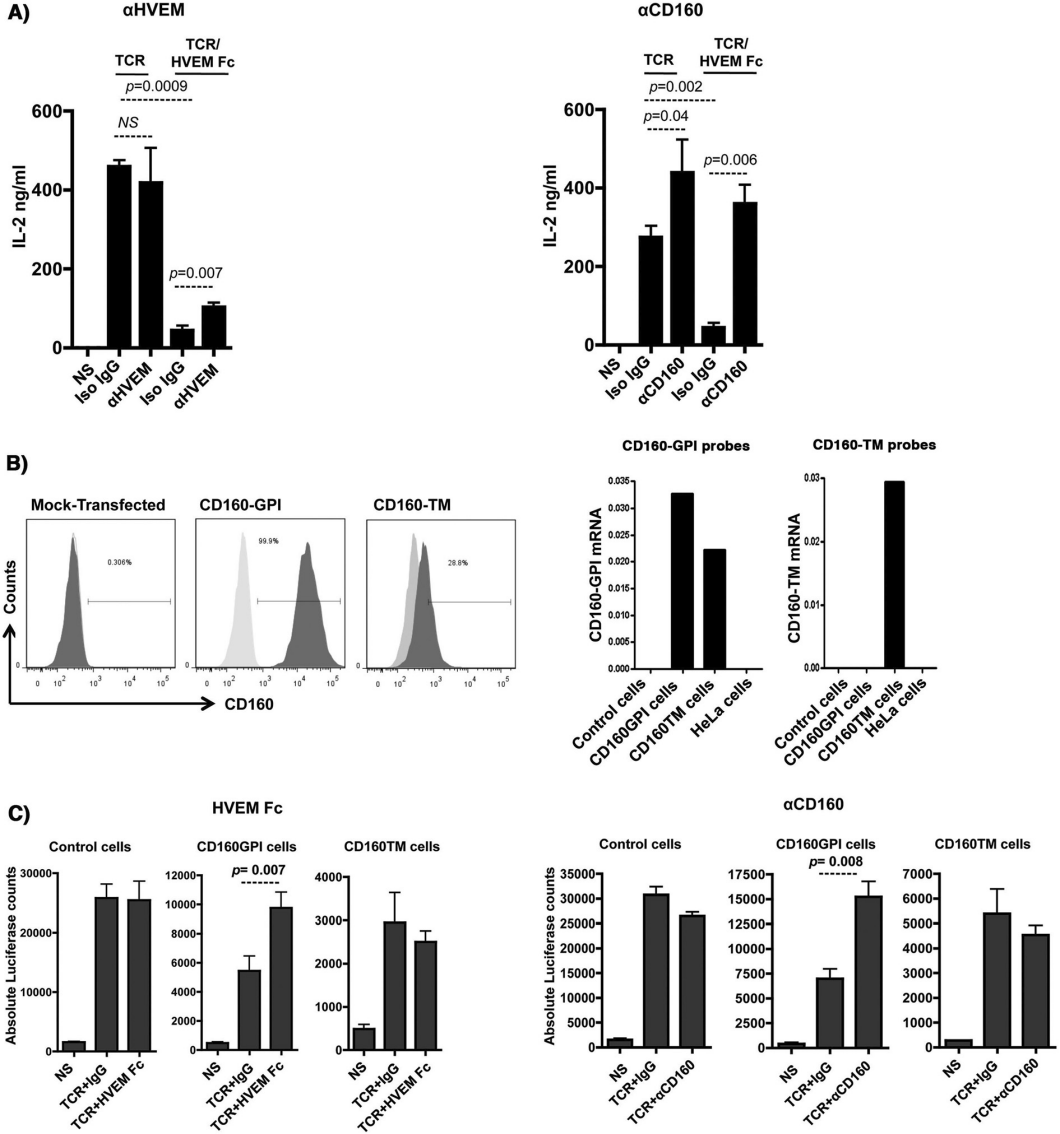
**Triggering of CD160-GPI is consistent with a positive co-stimulation role. A)** Triggering of primary CD4^+^ T-cells with either plate-bound anti-CD3 (1 μg/ml) and anti-CD28 (0.5 μg/ml) or anti-CD3, anti-CD28 and HVEM-Fc (0.2 μg/ml) in the presence or absence of either anti-HVEM (left panel) or anti-CD160 clone CL1-R2 (right panel). IL-2 was measured in the supernatant by ELISA at 24 h post stimulation. Iso-IgG represents the matched isotype control antibody. *P* values were determined by two-tailed paired *t* test (data from three independent healthy donors). **B) Left panels:** Surface expression of CD160-GPI and CD160-TM on Jurkat-NFAT-Luc cells stably-transfected with CD160 plasmids. Mock-transfected cells (light grey histograms in middle and right panels) were used to set the positive and negative gates for FACS. CD160-TM is weakly detected with CD160-GPI antibodies (BY55 clone). **Right panels:** Quantitative RT-PCR for CD160-GPI and CD160-TM isoforms in Jurkat cells over-expressing either CD160-GPI or CD160-TM, values are relative to the house-keeping GAPDH gene transcripts (One representative experiment, n = 2). Non-transfected Jurkat (control cells) and HeLa cells were used as additional negative controls for CD160 expression. The left graph represents results with a set of Taqman probes that were not isoform selective and hybrdize both CD160-GPI and CD160-TM to demonstrate similar RNA expression levels, whereas the right graph used a set of probes that were CD160-TM specific to confirm the exclusive expression of the different CD160 isoforms in the two cell lines. **C)** Simultaneous triggering of TCR and CD160 using magnetic Dynal beads coated with anti-CD3, anti-CD28 and either HVEM-Fc (left panels), CD160 monoclonal antibodies (right panels) or their matched IgGs. Cell activation was monitored by measuring the absolute luciferase counts. Control cells are original Jurkat-NFAT-Luc cells non-transfected with either of the CD160 isoforms. NS: non-stimulated. *P* values were calculated by non-parametric two-tail *t* test (Mann–Whitney).

An inhibitory assay with primary CD4^+^ T-cells was established whereby TCR and CD160 were simultaneously triggered in the presence or absence of specific antibodies to either HVEM or CD160 (CL1-R2 clone). CD4^+^ T-cells were isolated from total PBMCs of healthy donors and rested overnight to up-regulate CD160 as described earlier (expression was monitored by flow cytometry). As shown in Figure 3A (left and right panels), addition of HVEM-Fc significantly reduced IL-2 production from CD4^+^ T-cells that were stimulated with anti-CD3 and anti-CD28 antibodies for 24 h. Blockade of HVEM with specific anti-HVEM monoclonal Ab (Figure 3A left panel) partially restored IL-2 production from cells triggered with TCR/HVEM-Fc compared to treatment with matched isotype control Ab (*p* = 0.007). Similarly, treatment of TCR/HVEM-Fc triggered cells with CD160 specific monoclonal antibodies increased IL-2 production to reach levels equal to or higher than cells stimulated with TCR alone (Figure 3A right panel) (*p* = 0.006). Interestingly, IL-2 production by TCR-stimulated cells in the absence of the HVEM-Fc ligand was also enhanced by the CD160 mAb (*p* = 0.04). Meanwhile CD160 antibody had no impact on CD4^+^ T-cell activation in the absence of TCR stimulation, thus suggesting that the CD160 antibody-mediated enhancement of cell activation is TCR-dependent. These results are consistent with earlier reports showing that targeting CD160 with monoclonal antibodies may enhance TCR-mediated signaling in T-cells [32,33].

As we showed earlier, CD160 is expressed in at least two different isoforms on both CD4^+^ and CD8^+^ T-cells. To further gain insight into the functional roles of these two isoforms, we selectively and exclusively over-expressed cDNA clones of the alternatively spliced CD160-GPI or CD160-TM in Jurkat T-cell lines that also encode an NFAT-responsive luciferase reporter gene (Jurkat-NFAT-Luc) and assessed the functional impact of CD160 triggering by HVEM ligand and cognate mAbs. Figure 3B (left panel) shows FACS data for the different cellular clones that apparently represent high levels for CD160-GPI expression (up to 100%) and intermediate levels (up to 30%) for CD160-TM. The intermediate levels of CD160-TM detected by FACS reflects the difference of the CD160 BY55 monoclonal antibody, used in current phenotyping assays, in binding to the CD160-TM isoform relative to the CD160-GPI isoform [18]. To ensure similar levels of ectopic expression of the individual isoforms in the respective cell lines and to also confirm the absence of intrinsic CD160 expression, we quantified the CD160-GPI and CD160-TM mRNA transcripts ectopically expressed in each of these cell lines. Data presented in Figure 3B (right panel) showed that no intrinsic CD160 expression was detected in the non-transfected control Jurkat-NFAT-Luc cells. In contrast, Jurkat cells transfected with CD160-TM only expressed the full-length TM isoform transcripts whereas the Jurkat cells transfected with the CD160-GPI plasmid expressed the GPI transcripts. Two sets of Taqman probes were used in these studies and one set was not selective and hybridized both the short CD160-GPI and the full-length CD160-TM in the two respective cell lines and demonstrated similar RNA expression levels. Whereas the other set of probes were CD160-TM specific and confirmed the exclusive expression of the different CD160 isoforms in these two cell lines (Figure 3B right panels).

The effect of HVEM-mediated CD160 triggering on TCR activation was assessed by measuring the NFAT-responsive luciferase activity of Jurkat cells expressing either CD160-GPI or CD160-TM isoforms. Dynal Beads coated with anti-CD3, anti-CD28 and either HVEM-Fc or matched isotype control were used to perform these experiments. HVEM-Fc specifically activated Jurkat cells that expressed CD160-GPI, but not the TM isoform (Figure 3C, left 3 panels). Of note, enhancement of cell activation by HVEM-mediated CD160-GPI triggering was observed only when lower concentrations of anti-CD3 antibodies were loaded to the activator beads (Additional file 1A). To further ensure equal loading capacity for stimulating antibodies and ligands, activator beads were stained with secondary anti-mouse antibody and analyzed by FACS (Additional file 1B). Similar to HVEM-Fc-mediated CD160-GPI triggering, TCR co-stimulation with the CD160 monoclonal antibody CL1-R2 enhanced activation of Jurkat-CD160-GPI, but not Jurkat-CD160-TM (Figure 3C, right 3 panels). Identical results were also obtained with anti-CD160 clone 688327 (data not shown).

Altogether, the engagement of CD160-GPI, but not CD160-TM, by either HVEM-Fc or specific mAb enhanced the Jurkat T-cell activation as measured by the higher NFAT-responsive luciferase activity. The lack of any significant impact of HVEM-Fc on the CD160-TM isoform together with the positive co-stimulation mediated by HVEM-Fc triggering of CD160-GPI in the Jurkat assay suggested that the HVEM-Fc mediated inhibition of IL-2 production that we observed with primary CD4^+^ T-cells (Figure 3A) is likely mediated by HVEM interaction with BTLA, which is constitutively expressed on CD4^+^ T-cells (data not shown and [34]).

### CD160 and HVEM antibodies specifically enhance CD4^+^ T-cell responses to a recall antigen

**Figure 4:**
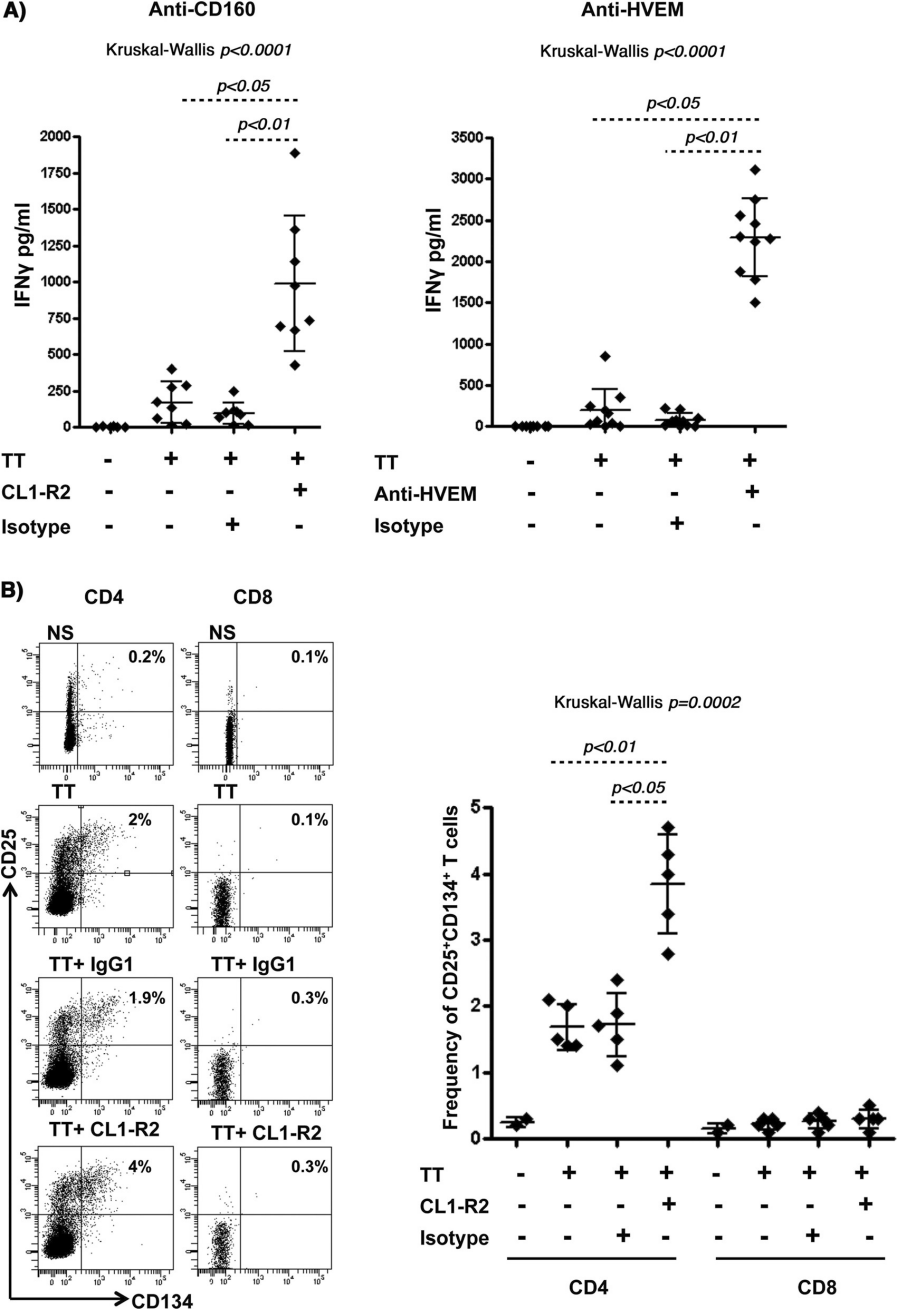
**CD160 and HVEM antibodies specifically enhance IFNγ production by CD4 T-cells in response to the Tetanus toxoid recall antigen. A)** IFNγ production by total PBMCs (1.5 × 10^6^ cells/ml) stimulated with 2.5 μg/ml of Tetanus toxoid in the presence or absence of anti-CD160 clone CL1-R2 mAb (**left panel**), HVEM pAb (**right panel**) or their matched isotype control Abs (n = 10). IFNγ was measured by ELISA from the supernatant following 5 days of stimulation. **B) Left panel:** Surface staining of cells using anti-CD3, anti-CD4, anti-CD8, anti-CD25 and anti-CD134 (OX40) analyzed by FACS (gating on CD3^+^ lymphocytes followed by gating on either CD4^+^ or CD8^+^ T-cells). **Right Panel:** analysis of the frequency of CD25^+^CD134^+^ double positive CD4^+^ and CD8^+^ T-cell populations (n = 5). *P* values were determined using the nonparametric Kruskal-Wallis and Dunn’s post-test.

As a first line high throughput assay capable of identifying antibodies that modulate antigen specific T-cell activation, we analyzed memory T-cell responses to Tetanus toxoid (TT) recall antigen. This assay allowed us to compare the potency of anti-CD160 (CL1-R2) mAb and the polyclonal anti-HVEM antibodies to enhance T-cell response. Both antibodies increased the production of IFNγ by PBMCs from healthy responders upon stimulation with suboptimal concentrations of TT (2.5 μg/ml) in a 5-day culture assay (Figure 4A). No IFNγ production was observed in the absence of antigenic stimulation (data not shown).

Tetanus toxoid is known to elicit a CD4^+^ T-cell response [35]. In order to confirm the assay specificity, we tested the CD4 response by monitoring the frequency of TT-specific CD4^+^ T-cells as determined by the surface expression of IL-2Rα (CD25) and OX40 (CD134). This method was previously shown to identify Ag-specific CD4^+^ T-cells without the need for HLA class II multimers [35]. Analogous to the experimental conditions described above, PBMCs from healthy responders were stimulated with the Tetanus antigen (TT) for 5 days in the presence or absence of anti-CD160 antibodies. As shown in Figure 4B, only CD4^+^ T-cells responded to TT-stimulation by up-regulating both CD25 and CD134 [the frequency of CD25^+^CD134^+^ DP cells increased from an average of 0.3% to 1.7% (n = 5)]. In contrast, no significant impact was observed on the CD8^+^ T-cell population. Interestingly, addition of CD160 mAb increased the frequency of CD25^+^CD134^+^ DP fraction to an average of 3.8% (n = 5), whereas no change in frequency was observed with isotype control antibodies. These results showed that CD160 and HVEM antibodies specifically enhanced memory CD4^+^ T-cell responses (both qualitatively and quantitatively) against a recall antigen upon re-stimulation.

### Combined targeting of CD160 and PD-1 enhances HIV-specific CD8^+^ T-cell proliferation

The impact of targeting CD160, with CD160-specific antibodies on HIV antigen-specific exhausted T-cells from HIV-infected subjects was studied *ex vivo* to evaluate its therapeutic potential. We first comprehensively screened HIV-1 epitopes by IFNγ ELISPOT to map the different T-cell responses to HIV-1 peptides from infected subjects. The primary objective of this comprehensive analysis was to determine the baseline responses to HIV-1 peptide stimulations from subjects with different categories/stages of disease and in turn to characterize the change in responses upon targeting CD160 and/or other key cell surface regulators, herein PD-1. Study samples were obtained from both cART-treated aviremic and cART-naïve viremic subjects, with one of four subjects having the protective HLA allele B27 (Table 1). As shown in Additional file 2, the breadth of *ex vivo* responses was higher in samples from the viremic subjects compared to samples from the successfully treated one. Samples from the HLA-B27 subject displayed the highest response values.

**Figure 5:**
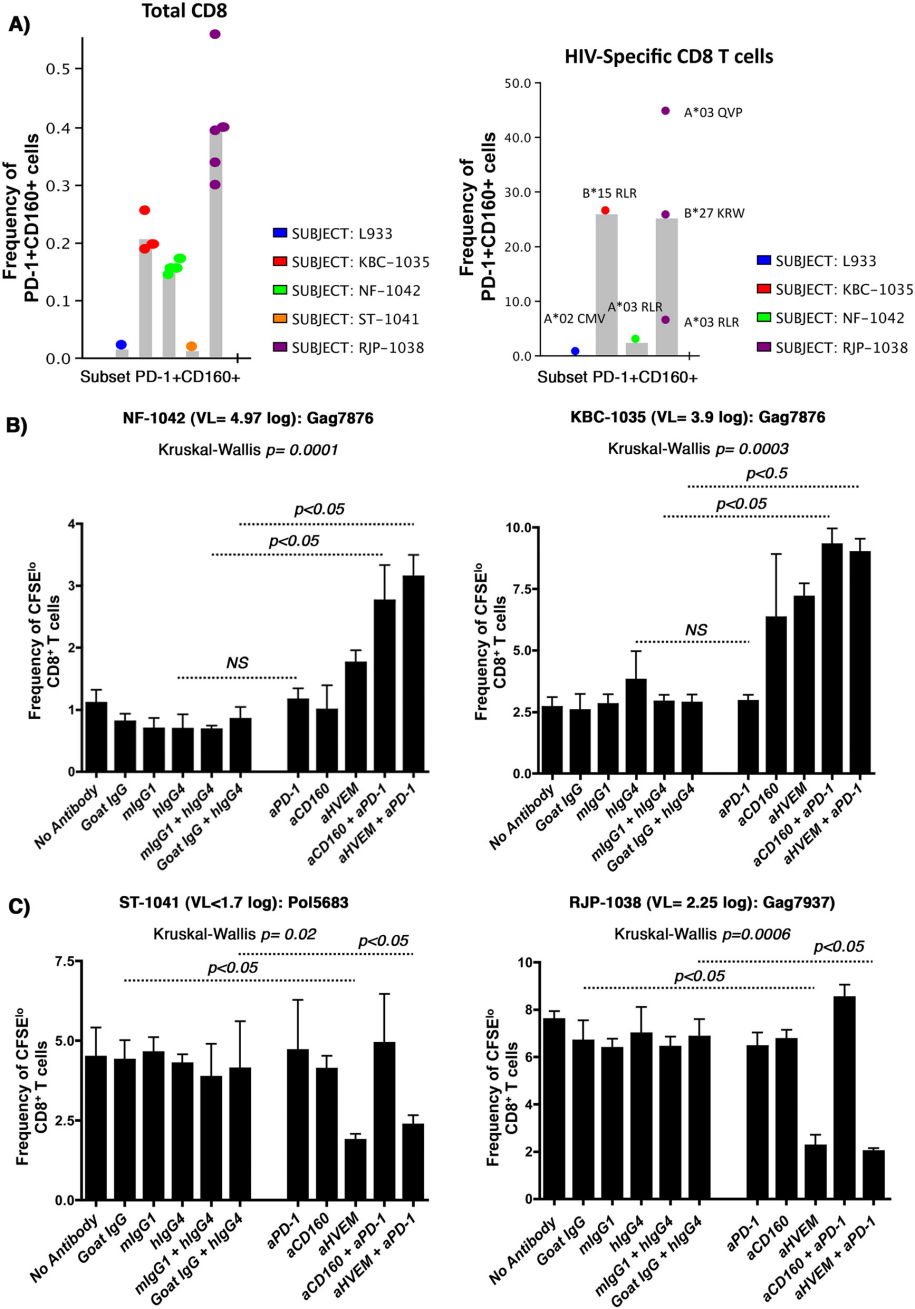
**Enhanced CD8** ^**+**^ **T-cell proliferation to antibody-mediated blockade of PD-1 in combination with either CD160 or HVEM antibodies. A)** Histogram summarizing the phenotypic analysis showing the frequencies of CD160^+^PD-1^+^ double positive population on total CD8 (**left panel**: each dot represents an independent staining from the same subject) and HIV-specific (**right panel**) T-cells from the four recruited study subjects. L933 represents the HIV-uninfected donor used as a control. HIV pentamers from each subject is annotated above each bar (**right panel**). Gating was done on CD3^+^ lymphocytes followed by gating on either total CD8^+^ T-cells or pentamer HIV-specific CD8^+^ T-cells. Note that we were unable to fold the peptides identified in the ELISPOT assay with HLA-restricted multimers from cART-treated subject. **B)** CFSE lymphoproliferation assays on total PBMCs from the viremic subjects NF-1042 and KBC-1035 gated on CD3^+^CD4^−^CD8^+^ T-cells. PBMCs stimulated or not with Gag7876 (restricted by HLA-B*1501 for KBC-1035, and HLA-A*0301 for NF-1042) in the absence or presence of blocking antibodies (4 replicates for each condition). **C)** PBMCs from ST-1041 and RJP-1038 stimulated or not with Pol5683 (restricted by HLA-A*11, A*03, A*68) and Gag7937, restricted by HLA-B*2705, respectively (4 replicates for each condition). *P* values were determined using the nonparametric Kruskal-Wallis and Dunn’s post-test.

Since CD160^+^PD-1^+^ double positive (DP) populations of HIV-1-specific CD8^+^ T-cells were previously shown to represent a highly exhausted cell subset [14], we measured the co-expression of CD160 and PD-1 on both total and selected antigen-specific cells based on the CD8^+^ T-cell epitopes defined by the IFNγ ELISPOT assay. As shown in Figure 5A, although the frequency of this DP population on total CD8^+^ T-cells was modest, the DP frequency was higher in CD8^+^ T-cells from HIV viremic subjects relative to the cART treated and virus-suppressed subject or healthy donors (**Left panel**). Most notably, the DP population comprised a relatively high proportion (3-45%, depending on the multimer used) of the HIV-specific CD8^+^ T-cells in the viremic subjects when compared to the A*02 CMV Ag-specific population from a HIV-uninfected donor (Figure 5A, right panel).

We further compared the effect of dual targeting of CD160 and PD-1 *versus* the dual targeting of HVEM and PD-1 [14] (at concentrations of 5 μg/ml for each individual antibody) on the functional restoration of HIV-specific CD8^+^ T-cell responses. The combination of CD160 and PD-1 specific Abs increased the frequency of proliferating HIV-specific CD8^+^ T-cells by 4.3- and 3.2-fold in samples from subjects NF-1042 and KBC-1035, respectively (Figure 5B, left and right panels, *p* = 0.04 for both). This was comparable to a combination of HVEM and PD-1 Abs that resulted in a 3.8- and 3-fold increase in HIV-specific CD8^+^ T-cell proliferation from these respective subjects (*p* = 0.03 for both). Lower levels of enhancement were observed when these antibodies were used individually, and these results are consistent with earlier observations in the LCMV mouse model that show enhancement of Ag-specific cell functions with anti-LAG-3 only upon combination with anti-PD-L1 [13]. Interestingly, we did not observe any significant enhancement of CD4^+^ T-cell proliferation in response to the p24 antigen in the presence of these antibodies (data not shown), which suggests that the observed functional enhancement was specific to CD8^+^ T-cells. Co-targeting of PD-1 with either CD160 or HVEM showed very low levels of enhancement when peptide pools specific to other infectious agents (CEFT: CMV, EBV, Influenza and Tetanus) were used as controls (Additional file 3A & B). Of note, no significant enhancement was obtained with the CD160 and PD-1 combined antibody treatment in samples from subjects with low viral load (ST-1041 and RJP-1038), whereas HVEM specific antibodies diminished the frequency of proliferating cells (compared to stimulation in the absence of antibody candidates) in these samples (Figure 5c). No activation-induced cell death (AICD) was observed with HVEM antibodies (data not shown).

## Discussion

CD160 belongs to the broad family of T-cell co-regulators. In our efforts to generate a screening assay for selecting antibody candidates with the capacity to block HVEM binding to CD160 and to functionally impact T-cell activation, we over-expressed the two known isoforms of CD160 (GPI and TM) in Jurkat cells harboring a luciferase reporter gene. HVEM ligand enhanced TCR-mediated activation only in cells expressing the CD160-GPI isoform and not the CD160-TM isoform. The lack of HVEM-mediated activation of CD160-TM may, in part, be due to the weak interaction between these proteins as suggested by our binding assays. However, as we could not confirm equal surface expression of CD160-TM, compared to CD160-GPI, due to the lack of CD160-TM specific antibodies, we cannot exclude the possibility that the low binding of HVEM-Fc to the CD160-TM expressing cells is due, at least in part, to a lower CD160-TM expression at the cell surface. Yet, similar levels of transcription were observed for both CD160-GPI and CD160-TM isoforms in the CHO-K1 cells, used for the binding assays, and in Jurkat cells, used for the functional assays. Furthermore, monoclonal and polyclonal antibodies to HVEM enhanced the binding of HVEM-Fc to the CD160-TM in the CHO-K1 cells, which suggests that CD160-TM was expressed to significant levels at the cell surface. Similar to antibody-mediated enhancement of HVEM-Fc binding to CD160, earlier observations were also reported for the binding of CD160 to MHC class I molecules [19]. The anti-MHC I monomorphic antibody W6/32 mAb enhanced interaction between cells expressing CD160 and cells expressing the class I molecules suggesting that ligand multimerization may promote binding to CD160-TM (20). However, multimerization of HVEM may not be the only possible mechanism to induce HVEM binding to CD160-TM as potential antibody-mediated changes in the HVEM protein conformation may also play a role The distinction between CD160-GPI and CD160-TM with regard to the need for HVEM multimerization or antibody-mediated conformational change might explain the lack of HVEM-mediated effect on Jurkat-CD160-TM with bead-bound monomeric HVEM-Fc fusion. How the MHC I or HVEM ligands localize/multimerize or change their conformational structure under physiological conditions in order to promote binding to CD160, requires further investigations. HVEM is expressed as a monomer and upon binding to the homotrimeric LIGHT forms a trimeric multimer [36,37]. Gonzalez *et al.* [37] suggest that BTLA is likely to bind to HVEM in the presence of LIGHT or LTα, whereby these latter receptors favor the formation of a trimeric HVEM. The regulation of HVEM association with CD160-TM through multimerization or conformational change and its impact on T-cell activation remains to be elucidated.

Triggering of CD160-GPI isoform over-expressed by the CD4^+^ Jurkat T-cell line with monoclonal antibodies in our study was consistent with a positive co-stimulatory role. Similarly, CD160 stimulation was previously shown to enhance CD3-induced activation and proliferation of peripheral blood CD160^+^ T cells [33] and also CD4^+^CD160^+^ T cells isolated from inflammatory skin lesions [32]. Though these results are in accordance with earlier reports that used the anti-CD160 CL1-R2 (IgG1) or the BY55 (IgM) [33] clones, they contrast with recent work by Cai *et al*., [23] showing that triggering of CD160 on primary CD4^+^ T-cells with the CD160 monoclonal antibody 5D.10A11 inhibits cell activation and cytokine production. These apparently discordant observations suggest that CD160 may differentially regulate either activating or inhibitory signaling pathways, which may depend on the type/clone of antibody or cognate ligand used to engage the target. Furthermore, the existence of two isoforms of CD160 (GPI and TM) in CD4^+^ and CD8^+^ T-cells with a possible differential expression and regulation of ligand binding may also account for the divergent reports on CD160 functions as the selectivity of 5D.10A11 antibody [23] for the various CD160 isoforms and the resulting effect on TCR signaling have not been characterized. Of note, in our Jurkat-NFAT-Luciferase assay with CD160-TM expressing cells, HVEM-Fc did not elicit either a negative or positive effect and may reflect a requirement for HVEM multimerization or induced conformational changes to promote CD160-TM binding.

Our study also showed that the GPI isoform was up-regulated on rested T-cells (both CD4^+^ and CD8^+^) *ex vivo* likely due to the culture conditions. This apparent up-regulation of CD160 on resting cells and the contribution of *ex vivo* culture conditions such as the use of human serum require more investigation. Yet CD160 was down-regulated by TCR activation, which indicates that expression of CD160 on primary T-cells is more complex than initially thought. CD160-GPI is likely to undergo receptor shedding upon T-cell stimulation similar to the previously described mechanism for CD160 on NK cells stimulated with IL-15 [38]. Although CD160-GPI and CD160-TM share the same extracellular domains, the GPI isoform does not contain a transmembrane domain. The two isoforms have differential binding characteristics for CD160 antibodies [18] and they may also differ in their signaling capacity. The presence of these two isoforms of CD160 and their potential differential expression in T-cells requires further studies, particularly in the context of immune exhaustion. Indeed, our results showed that HVEM antibodies function differently in *ex vivo* T-cell assays on samples isolated from HIV-infected subjects with higher viral loads compared to aviremic subjects. These antibodies restore HIV-specific CD8^+^ T-cell proliferation in lymphocytes isolated from viremic subjects, but in contrast dampen the response in CD8^+^ T-cells from aviremic subjects. This difference may be related to potential differential expression of the CD160 isoforms in viremic and aviremic subjects, meanwhile assuming that CD160-TM mediates a negative regulatory role in this context. Another potential setting could also be that the anti-HVEM antibodies may enhance binding of HVEM to the negative regulator BTLA that might be differentially expressed in aviremic *versus* viremic subjects. However these different regulatory mechanisms need more investigations.

Our functional analyses suggest that a pharmacologic effect in HIV viremic subjects may be elicited through the co-targeting of both CD160 (through Ab-mediated activation) and PD-1 (through Ab-mediated blockade). In one notable instance where the CD160^+^PD-1^+^ DP HIV-specific CD8^+^ T-cell subset was significantly higher in the HLA-B*2705 chronic infected subject compared to the HIV-uninfected control, the combined targeting of CD160 and PD-1 did not enhance response to HIV antigens. However, this subject had the largest breadth and magnitude of response to HIV peptides in agreement with earlier reports associating the HLA-B*2705 allele with protection from disease progression in HIV [39,40] and virus clearance in HCV [41]. In contrast to the B*2705 subject, the successfully treated subject showed low frequencies of the CD160^+^PD-1^+^ DP HIV-specific CD8^+^ T-cell, which is likely associated with low levels of viremia (less than 40 RNA copies/ml) and consequently reduced immune activation [14]. Similar to the B*2705 subject, combined targeting of CD160 and PD-1 in the successfully treated subject did not enhance HIV-specific T-cell proliferation and surprisingly, HVEM antibodies decreased cell proliferation likely by enhancing binding of HVEM to CD160-TM or BTLA [28,29]. This finding shows that functional T-cells may lose their capacity to proliferate and suggest that chronicity of infection and viral load levels may be used as predictive markers to identify patients who may benefit from immunotherapeutic intervention that target immune checkpoint molecules.

## Conclusions

In this study we used *in vitro* and *ex vivo* cellular assays to evaluate the targeting of CD160, relative to HVEM, as a co-target with PD-1 in immunopotentiating a response to HIV infection. Antibodies against CD160 and PD-1, used in combination, significantly enhanced HIV-specific CD8^+^ T-cell proliferation in response to HIV antigens from viremic subjects but showed no impact on CD8^+^ T-cell response from aviremic subjects. Therapeutic immunopotentiation through the specific targeting of negative and positive immune regulators on T-cells represents an interesting approach to complement current treatment regimens in HIV infection. To further our understanding on the HVEM/BTLA/LIGHT/CD160 network during disease, and to identify new correlates or predictive biomarkers in patients who may benefit from the combined Ab treatment with other targets, it would be interesting to analyze the differential expression of these molecules, including the two isoforms of CD160, in a longitudinal study that spans acute, chronic and treatment phases.

## Additional files

Additional file 1:
**Stimulation of Jurkat-CD160-GPI with decreasing concentrations of anti-CD3 in the presence or absence of HVEM-Fc.** **A)** Dynal Beads (4 × 10^7^ beads) were loaded with 80, 40, 20 or 10 ng of anti-CD3, a fixed concentration of anti-CD28 (1 μg) and 3.2 μg of either HVEM-Fc or the Isotype control antibody IgG2a. Stimulation was performed for 24 h at a ratio of 4 beads/cell. *P* values were calculated by non-parametric two-tail *t* test (Mann–Whitney). **B)** A representative Loading control for activator beads (set #4: 10 ng of anti-CD3) monitored by FACS. Beads loaded with anti-CD3, anti-CD28 and either HVEM-Fc or Isotype control were stained with the secondary antibody Goat anti-mouse (GAM-FITC). Additional file 2:
**Breadth of responses to HIV-1 clade B consensus peptides measured by IFNγ ELISPOT assay. Absolute numbers of recognized peptides to HIV-1, calculated as the sum of all responses to peptides from the same protein.** Responses are derived from four HIV-1 infected subjects described in Table 1. A larger breadth was observed in the B*027-expressing subject. Additional file 3:
**CFSE lymphoproliferation assays on total PBMCs from the four subjects stimulated with the control peptide pools CEFT (4 replicates for each condition).** *P* values were generated using the nonparametric Kruskal-Wallis and Dunn’s post-test. * Represents a significant *p* value <0.05.
