# Supplementary figures and images for: CD160 isoforms and regulation of CD4 and CD8 T-cell responses
Source: J Transl Med. 2014 Sep 2;12:217. doi: 10.1186/s12967-014-0217-y (PMC4163173; doi:10.1186/s12967-014-0217-y)

A)

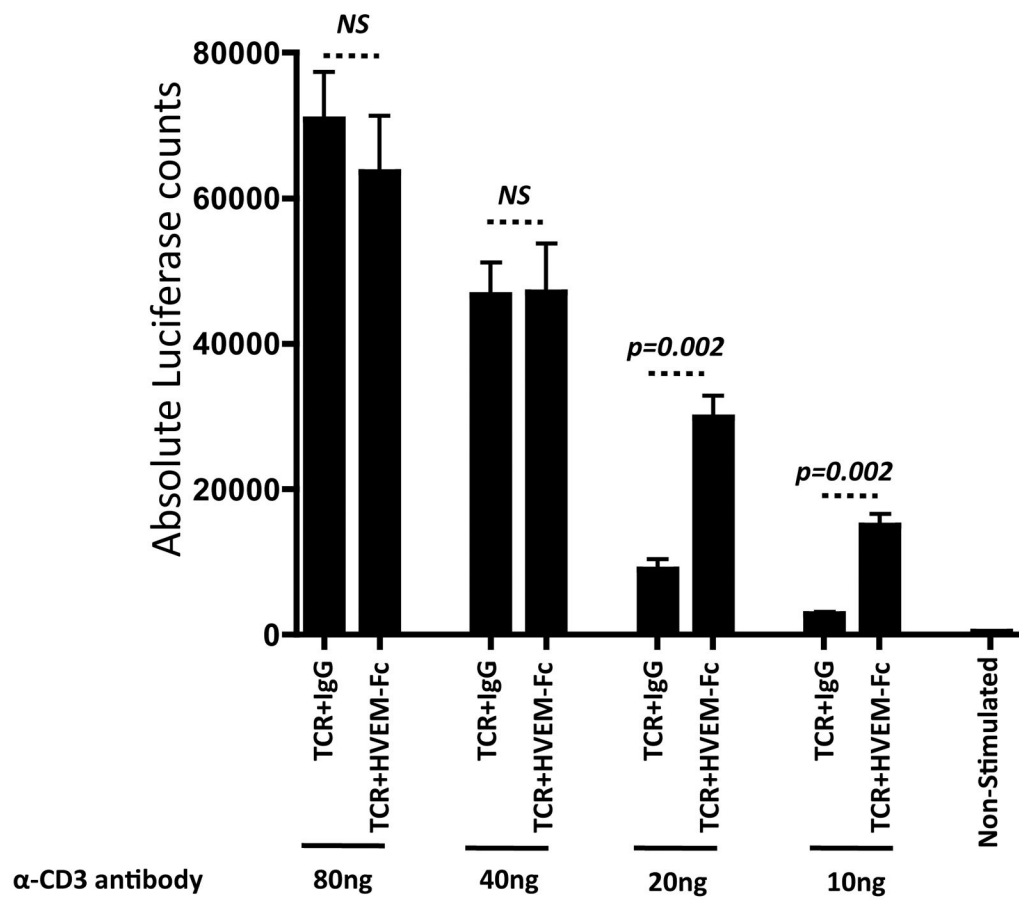

B)

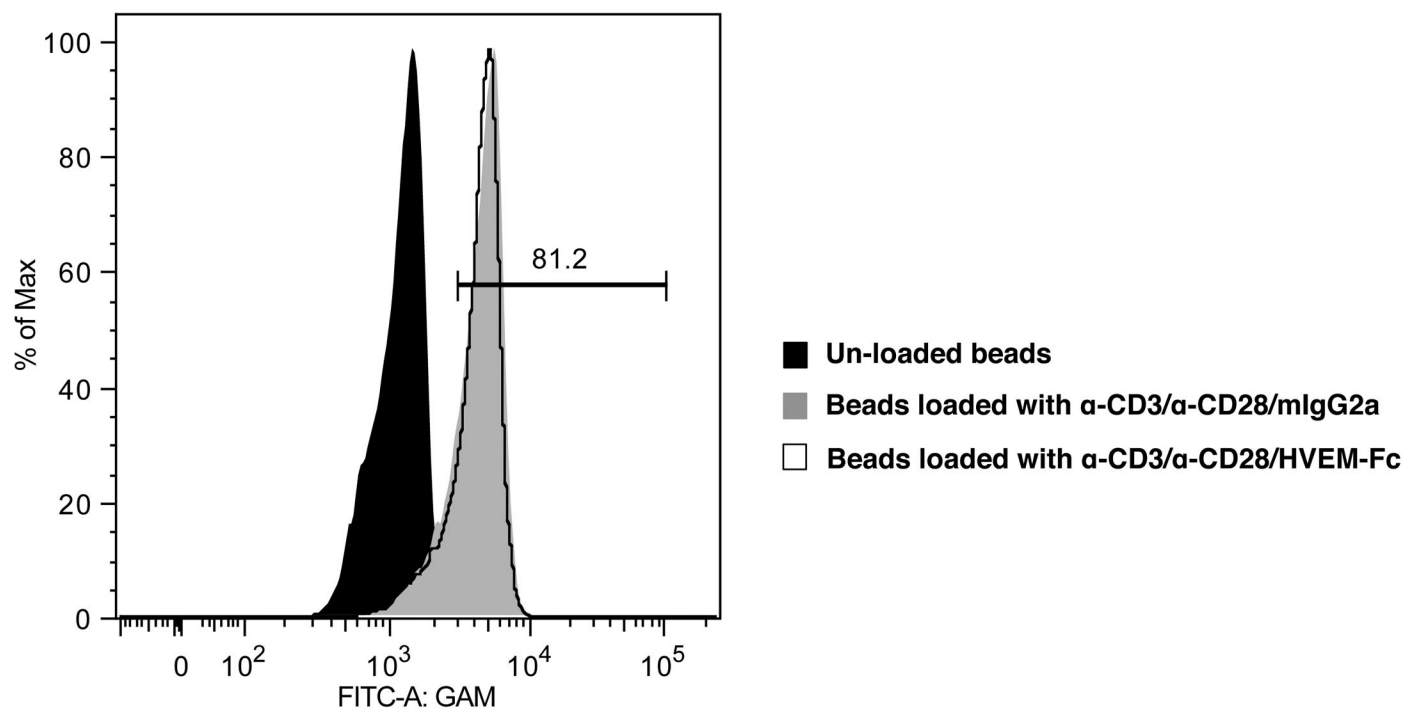

Supplement: Additional file 1: — Stimulation of Jurkat-CD160-GPI with decreasing concentrations of anti-CD3 in the presence or absence of HVEM-Fc. A) Dynal Beads (4 × 10^7^ beads) were loaded with 80, 40, 20 or 10 ng of anti-CD3, a fixed concentration of anti-CD28 (1 μg) and 3.2 μg of either HVEM-Fc or the Isotype control antibody IgG2a. Stimulation was performed for 24 h at a ratio of 4 beads/cell. P values were calculated by non-parametric two-tail t test (Mann–Whitney). B) A representative Loading control for activator beads (set #4: 10 ng of anti-CD3) monitored by FACS. Beads loaded with anti-CD3, anti-CD28 and either HVEM-Fc or Isotype control were stained with the secondary antibody Goat anti-mouse (GAM-FITC). [file 12967_2014_217_MOESM1_ESM.pdf]

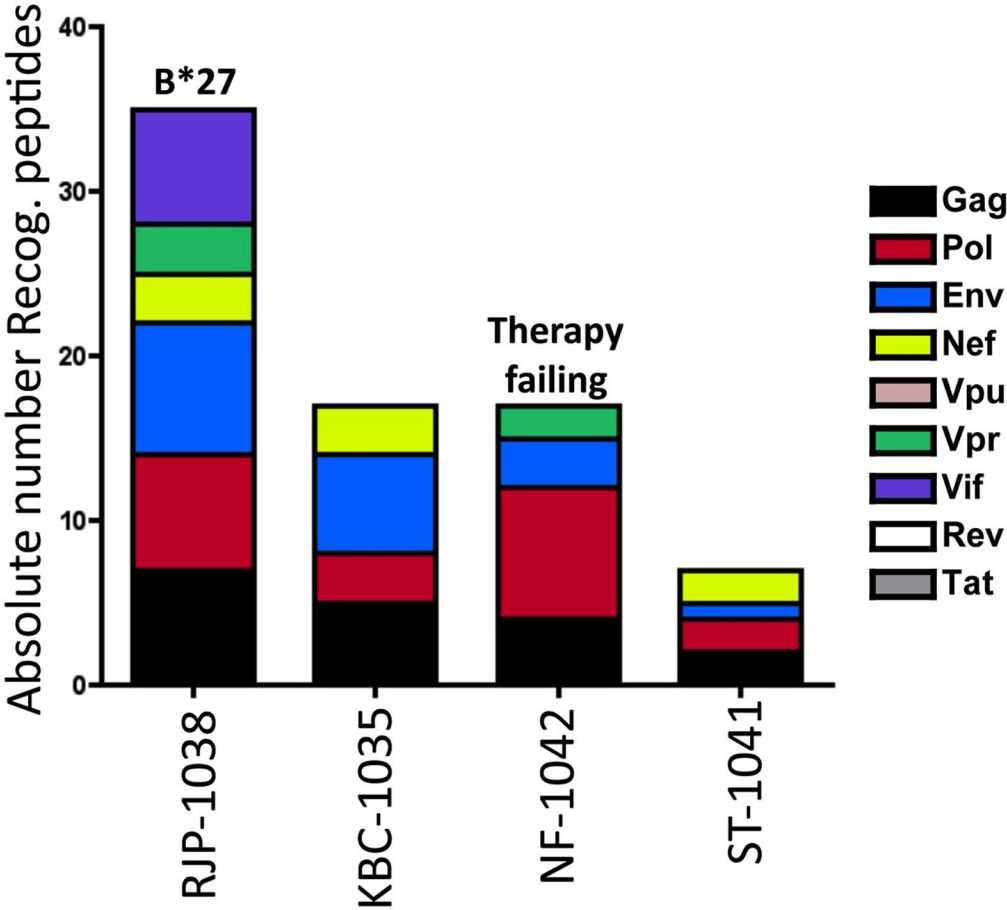

Supplement: Additional file 2: — Breadth of responses to HIV-1 clade B consensus peptides measured by IFNγ ELISPOT assay. Absolute numbers of recognized peptides to HIV-1, calculated as the sum of all responses to peptides from the same protein. Responses are derived from four HIV-1 infected subjects described in Table 1. A larger breadth was observed in the B*027-expressing subject. [file 12967_2014_217_MOESM2_ESM.pdf]

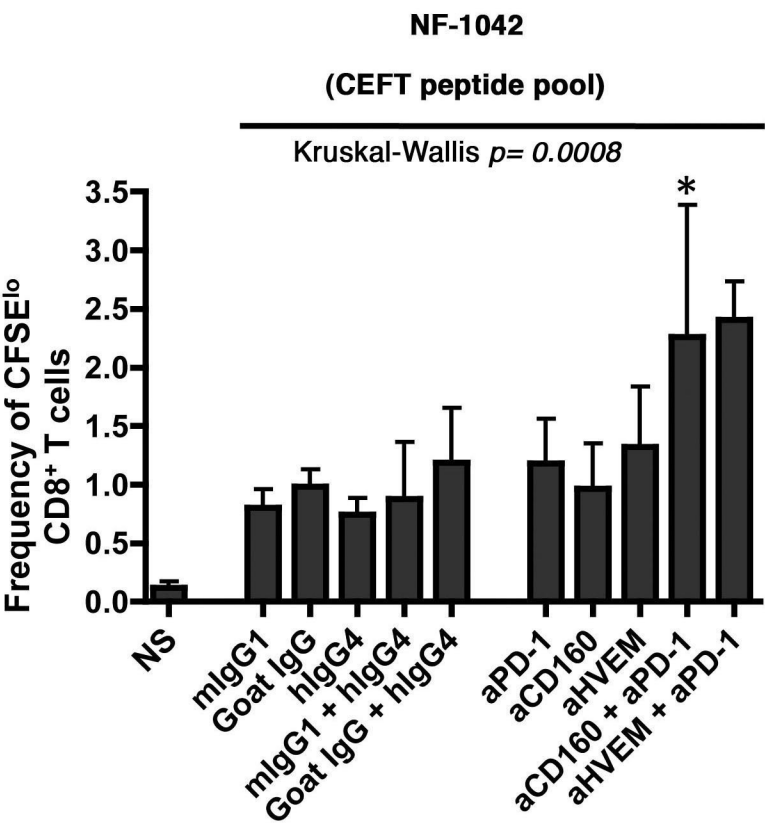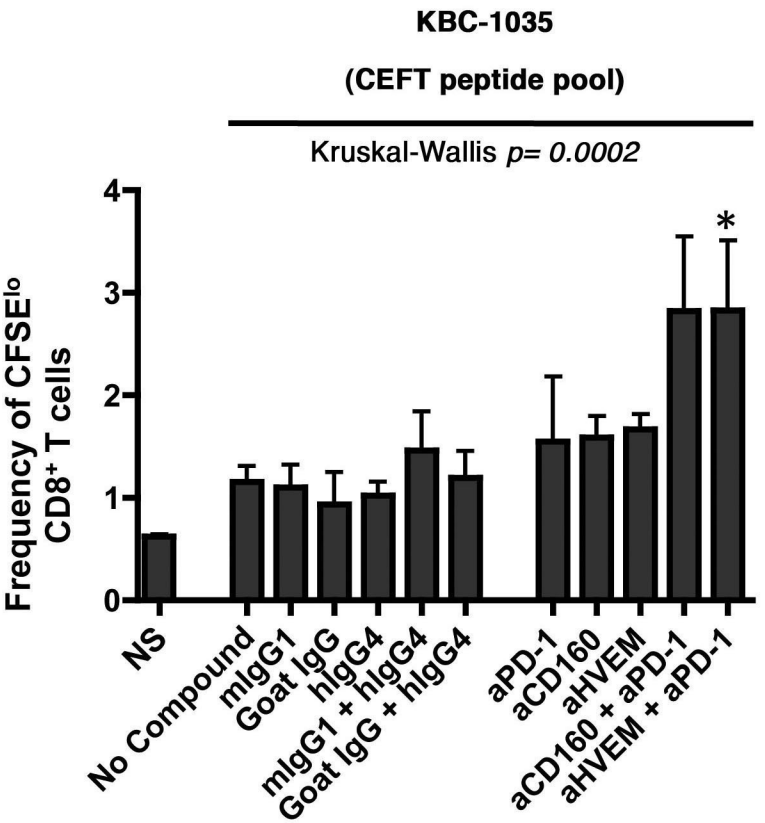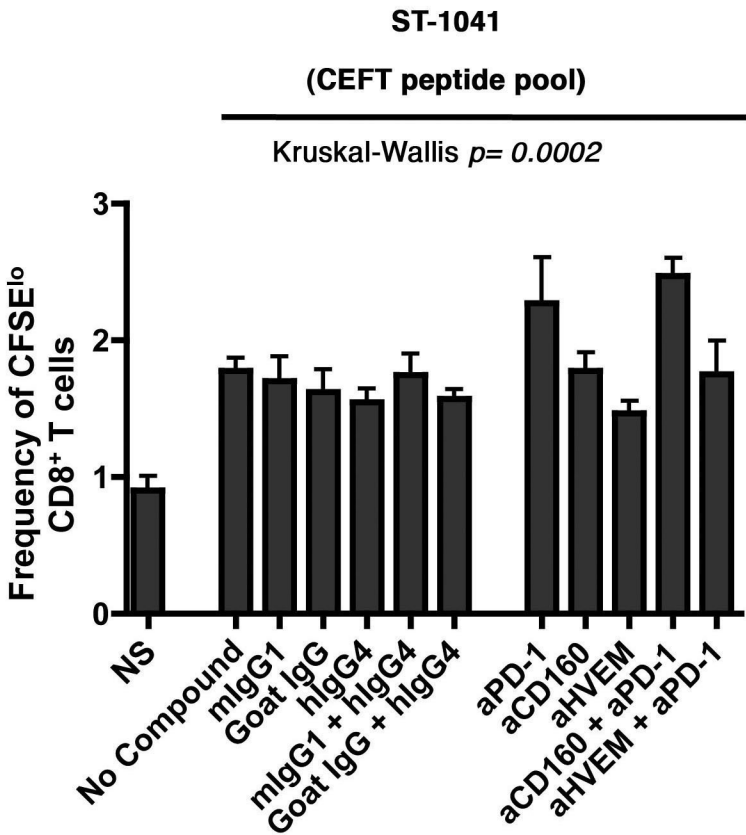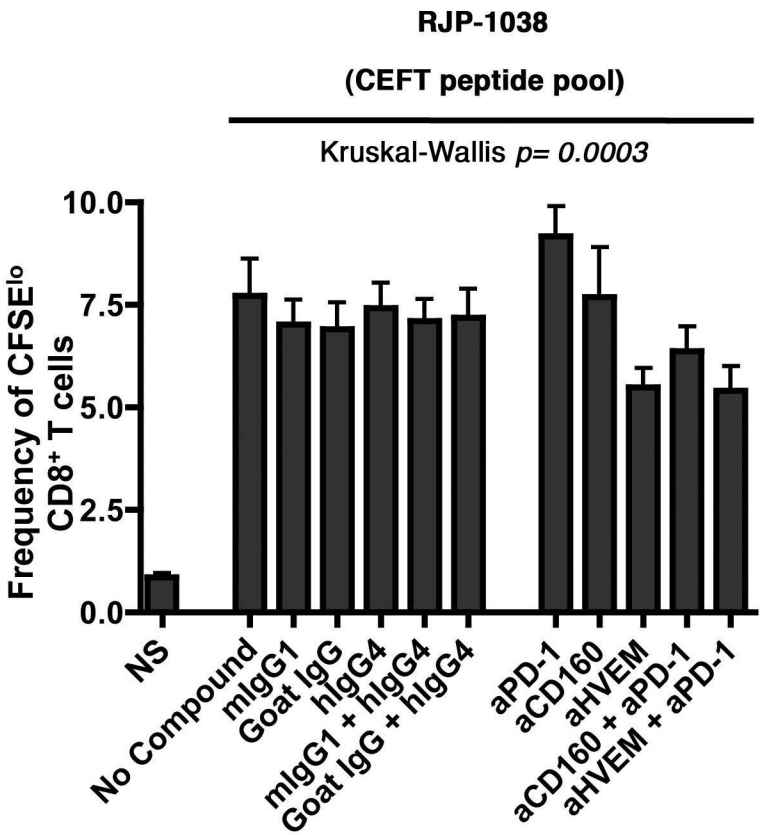

Supplement: Additional file 3: — CFSE lymphoproliferation assays on total PBMCs from the four subjects stimulated with the control peptide pools CEFT (4 replicates for each condition). P values were generated using the nonparametric Kruskal-Wallis and Dunn’s post-test. * Represents a significant p value <0.05. [file 12967_2014_217_MOESM3_ESM.pdf]
